# Off-label medicine use in children and adolescents: results of a population-based study in Germany

**DOI:** 10.1186/1471-2458-13-631

**Published:** 2013-07-03

**Authors:** Hildtraud Knopf, Ingrid-Katharina Wolf, Giselle Sarganas, Wanli Zhuang, Wolfgang Rascher, Antje Neubert

**Affiliations:** 1 Department of Epidemiology and Health Monitoring, Robert Koch Institute, General-Pape-Str. 62-66, 12101, Berlin, Germany; 2 Institute of Clinical Pharmacology and Toxicology, Charité Universitätsmedizin Berlin, Charitéplatz 1, 10117, Berlin, Germany; 3 Department of Paediatrics and Adolescent Medicine, Paediatric Clinical Study Center, University Hospital Erlangen, Loschgestr. 15, 91054, Erlangen, Germany

**Keywords:** Off-label, Children, Paediatric, KiGGS study, Under-dosing

## Abstract

**Background:**

Population-based self-reported data on off-label medicine use independent from health care provisions are lacking. The purpose of this study is to investigate off-label medicine use in children and adolescents in Germany in a non-clinical setting and to identify prevalence, determinants and spectrum of off-label medicine use.

**Methods:**

Data were obtained from the German Health Interview and Examination Survey for Children and Adolescents (KiGGS) conducted by the Robert Koch Institute (2003–2006). 17,450 randomly selected children aged 0–17 years took part in the drug interviews. Of those, 8,899 took at least one medicine during the 7 days preceding the interview. Off-label medicine use was defined as the discrepancy between actual use and the intended use described in the summary of product characteristics. Off-label medicine use was stratified into off-label indication, off-label age, off-label over-dosing, and off-label under-dosing.

**Results:**

The prevalence rate of off-label medicine use among those who used medicines amount of is 40.2%. The prevalence rate is significantly higher in boys (41.4%), in children aged 3 to 6 years (48.7%), without migration background (40.9%), with high social status (42.5%), living in small (42.0%) and medium sized cities (41.6%), and with a poor parents rated health status (41.7%). 12,667 preparations (attributable in respect to off-label use) were taken by 8,899 children. 30% of the medicines have been used off-label. Off-label medicine use was highest in preparations of the ATC-class “C00 Cardiovascular System”. In all origins of medicine, all age groups and all ATC-classes under-dosing was the most frequent reason for off-label medicine use.

**Conclusions:**

There is a considerable level of self-reported off-label medicines use in the general paediatric population. Further investigations are needed to examine in how far off-label medicine use is based on lack of knowledge or on empiricism in paediatric pharmacotherapy. Attention also needs to be paid to under-dosing which potentially exposes drug users to risks of side effects without the benefit of a therapeutic effect. Clinical trials for licensing of paediatric medicines, education of health care professionals, but also of parents and carers are needed to ensure the rational use of medicines.

## Background

Off-label medicine use among children and adolescents constitutes an important public health issue as the effects and potential health risks may be unpredictable. Pioneering work in this area was conducted already in the year 1953 by the paediatrician F. Dost [1] who stated, that children differ pharmacokinetically from adults, and medication for adults cannot simply be administered in smaller doses. As children differ in their absorption, distribution, metabolism, and excretion of medicine, they have special needs with respect to their diseases and the dosing of medicines and they often require special formulations to permit administration of adequate doses. Particularly young children are unable to swallow tablets and are vulnerable to the taste of medicine. However, many medicines are not specifically developed for children. Among medicines which were newly licensed by the European Medicine Agency (EMA) between 1995 and 2005 only one third was specifically licensed for children [2]. There is hope that this situation will improve with the Paediatric Regulation [3] which came into force in 2007 and which requires companies to develop a paediatric investigation plan (PIP), while granting incentives once a license for paediatric use has been obtained. Nevertheless, up to date there is still a high use of off-label medicine in children. National and international studies report a wide range of prevalence rates of off-label medicine use in children and adolescents, reaching from 3.2% to 80% [4-18].

Studies reporting the extent of off-label medicine use in the paediatric population are based on various data sources, such as prescription-, health care insurance-, or secondary care data. However, to our knowledge there are no representative self-reported population based data available. With the National Health Survey for Children and Adolescents (KiGGS) [19] for the first time representative self-reported data are available for analyzing the exposition to off-label medicine use in the general population of children and adolescents in Germany.

The present study utilizes population representative epidemiological data of KiGGS to investigate off-label medicine use in children and adolescents in a non-clinical setting and independent from any health care provisions. The objectives of this study are to assess the prevalence rates, determinants and spectrum of off-label medicine use. Furthermore, the spectrum of off-label medicine use will be analyzed with respect to prescribing status as well as in relation to the substance group.

## Methods

### Data collection and study population

Data for this study were collected within the German Health Interview and Examination Survey for Children and Adolescents (KiGGS) formally conducted by the Robert Koch Institute between May 2003 and May 2006. The survey’s target population consisted of all non-institutionalized children and adolescents aged between 0 and 17 years living in Germany. The design, sampling strategy and study protocol have been described elsewhere in detail [18]. Briefly, two-stage sampling procedures were applied. In the first stage, a sample of 167 German municipalities (112 in the former West Germany, 55 in the former East Germany) was drawn which was representative of municipality sizes and structures in Germany. Stratified by sex and age, random samples of children and adolescents between the ages of 0 and 17 years were then drawn from local population registries in proportion to the age and sex structure of Germany’s child population, including children and adolescents with a foreign nationality. Children, who were at time of survey, in hospital or medical nursing institutions were excluded. Among the selected study participants the response rate was 66.6%. A non-responder analysis was carried out to secure the representativeness of the sample [19].

The final sample included 17,641 children and adolescents (8,985 boys, 8,656 girls). All participants were invited to the study centres and asked to take part in the following data collection methods: to complete self-administered questionnaires, to participate in computer-assisted structured interviews administered by physicians, to undergo laboratory and other tests, and to take part in physical medical examinations. Part of the physical medical examinations was the documentation of standardized anthropometric measures, of those, only the measurement of body weight and height were relevant for this study. Other relevant data for this study were collected via the self-administered questionnaires (socio-economic status, migration background, parent-rated subjective health status of their children), and the personal computer assisted interviews administered by physicians (drug-use interview).

191 study participants did not take part in the drug-use interview and were excluded, resulting in a basic population of 17,450 (8,880 boys, 8,570 girls). Of those only participants with at least one medicine usage (n = 8,899) were included in our analyses of off-label medicine use.

The survey was approved by the Ethics Committee of the Virchow Hospital, Humboldt University Berlin and federal data-protection officials. Written, informed consent was obtained prior to each interview and examination from the children’s parents and the children themselves if they were aged ≥ 14 years.

### Definition of health-related and socio-demographic variables

While written, informed consent for participation in interviews and examinations was obtained from all parents/guardians as well as from children, aged ≥ 14 years, children could already at the age of 11 years or older fill in a standardised child questionnaire. The parent’s questionnaires were completed by the parents/guardians for all children, also for adolescents who had completed the standardised child questionnaire. These questionnaires were used to collect e.g. information on socio-economic data, family background, parent-rated child health status, and health-related living conditions. A family socio-economic status (SES) score was computed based on information obtained from both parents (if possible). The SES-score included information on the educational level, vocational status and the family net income [20]. After computing a total score from the above mentioned items with a minimum of 3 and a maximum of 21 points, study participants were assigned to one of three status groups depending on their individual score [20]. Participants were thus assigned to low, middle or high SES. Family immigration status was assessed using information on nationality, country of birth, and year of immigration of both parents. Study participants were classified as having an immigration background if they themselves were immigrants from another country and at least one parent was not born in Germany, or if both parents were immigrants or not of German nationality [21]. Living in East or West Germany as well as living in rural or urban areas was assessed by items concerning the place of residence. Depending on the number of inhabitants, communities were distinguished as rural (< 5,000), small-sized urban (5,000 - < 20,000), medium-sized urban (20,000 - < 100,000), and large city (100,000 and more). Parents rated the general health status of their children as ‘’excellent’, ‘good’, ‘moderate’, ‘bad’ or ‘very bad’. Because of the small numbers, within the last three categories they were summarised as ‘moderate/bad/very bad’.

### Assessment of medicine use

The use of any medication in the last seven days, including prescribed and OTC drugs was assessed in a face-to-face interview. The interviews were conducted by physicians using a standardized computer-assisted personal drug use interview tool [22]. All survey participants and parents were asked in advance to bring prescriptions or original packages to the examination site to facilitate the investigation and verification of drug use. Drug use was assessed by the following question:

‘Has your child taken any drugs in the last seven days? Please also mention the use of any ointments, liniments, contraceptive pills, vitamin and mineral supplements, medicinal teas, herbal or homoeopathic medicines’.

Details on every drug mentioned were collected such as brand name, indication, daily dose, route of application, frequency of intake, origin of the drug and duration of use.

Specific Anatomical Therapeutic Chemical codes (ATC-codes) were assigned to all reported medications, and International Classification of Diseases-10. Revision codes (WHO ICD-10 codes) to the conditions for which the medications were taken.

Off-label medicine use was stratified by self-medicated and prescribed preparations. Self-medicated preparations were defined as medicines that were either bought OTC or obtained from other sources. Prescribed medicines were preparations that were prescribed by a physician or by a non-medical practitioner.

### Data processing

Based on information provided by the study participants about the usage of each medicine (brand name, indication, dose, and frequency of use) and on characteristics of the study participants (age, height, body weight) a comparison was made with the summary of product characteristics (SPC) or patient information leaflet. If this comparison showed a discrepancy between actual and licensed usage the utilization was considered as off-label medicine use. Off-label medicine use was classified into the following strata:

1. Off-label indication

2. Off-label age

3. Off-label over-dosing

4. Off-label under-dosing

5. In-label

6. Not-attributable

Off-label indication was defined as the discrepancy between self-reported indication and the indication of the SPC. The same applies to the other chosen categories (off-label age, off-label over-dosing, off-label under-dosing). No off-label medicine use (in-label) was assigned if self-reported and SPC information was identical. If the available data about the medicine used was imprecise, the preparation was classified as not-attributable. Each product could only be classified in one category. If the product was off-label in more than one category off-label indication had highest priority followed by age, over-dosing, and under-dosing, respectively.

SPC and patient information leaflet were primarily taken from the German drug dictionary “Gelbe Liste” [23]. If this information was insufficient, “Rote Liste” [24], a different German drug dictionary, was consulted alternatively. If the information was still insufficient an internet research was conducted and if this was also unsuccessful the product was classified as “not-attributable”.

Children with off-label medicine use were boys and girls who took at least one off-label preparation. Children with in-label medicine use were defined as the total number of children and adolescent who took all their medicines in-label. Boys and girls exclusively using preparations that could not be allocated were defined as children with not-attributable off-label medicine use.

### Quality control

To determine the accuracy of the off-label assessment a 5% random sample (n = 738) was re-assessed by an external reviewer with previous experience in off-label medicine use in children. Interrater reliability between the original assessment and the second reviewer for the random sample was calculated using Kappa Statistics.

### Statistical analysis

In order to achieve a representativeness of the survey population a weighting factor was computed and used to adjust for deviations in demographic characteristics. This was a necessary step as the sampling was based on a two stage procedure (see chapter “Methods”, sub-chapter “Data collection and study population”). Basis for the adjustments was a comparison of the survey population with the official population statistics.

Descriptive statistics (proportions and 95% confidence intervals) were calculated to estimate prevalence rates of overall drug use, in-label- and off-label medicine use according to sex, age, region of residence, urbanity, migration background, social status, and parents’ rated health status.

In a multivariate logistic regression model odds ratios (ORs) and 95% confidence intervals (95% CIs) were estimated. The dependent variable was off-label versus in-label medicine use. In the logistic regression model children exclusively using not-attributable preparations were excluded. All variables of the descriptive calculation were included in the model as potentially determining factors.

Group differences were considered statistically significant if the 95% CIs of two rates did not overlap or the p-values were ≤ 0.05. All statistical analyses were performed using SPSS statistical software (release 20.0). In order to adjust for sample clustering effects, the SPSS complex samples module was used for all analyses.

## Results

### Prevalence and determinants of off-label use

A total of 17,450 children and adolescents participated in the drug interview. 8,899 boys and girls use at least one medication in the last seven days and thus constitute our study population.

Prevalence of medicine use is shown in Table 1. Girls have a significantly higher prevalence rate of drug use than boys. With increasing age the prevalence of medicine use is decreasing. Until the age of 2 years the medicine use is significantly higher than in all other age groups. There are no differences according to region of residence or according to urbanity. Children with a migration background or from families with a lower social status are significantly less often medicine user. Looking at the parents’ rated health status we find that children with a better health status use significantly less medication.

**Table 1:** Prevalence and determinants of off-label medicine use, KiGGS 2003-2006

	**Children with drug use (n = 8,899)**			**Children with off-label medicine use of ≥ 1 preparation (n = 3,610)**		**Children with excl. in-label medicine use (n = 4.334)**		**Children with excl. not-attributable medicine use (n = 955)**		**Children with off-label vs. excl. in-label medicine use (n = 7,808)**		
	**n**	**%**	**95% CI**	**%**	**95% CI**	**%**	**95% CI**	**%**	**95% CI**	**OR**	**95% CI**	**p-value**
Total	8,899	50.8	(49.5-52.2)	40.2	(38.8-41.5)	48.7	(47.3-50.2)	11.1	(10.3-12.0)			
Sex												.015
Boys	4,362	48.7	(47.2-50.3)	41.4	(39.7-43.1)	46.2	(44.5-47.9)	12.4	(11.3-13.6)	1.13	(1.03-1.25)	
Girls	4,537	53.1	(51.5-54.7)	38.9	(37.1-38.8)	51.2	(49.3-53.2)	9.8	(8.9-10.9)	1(Referenz)		
Age group												< .001
0 - 2 years	2,089	74.9	(72.9-76.9)	42.4	(39.8-45.1)	53.1	(50.3-55.9)	4.5	(3.5-5.8)	1.45	(1.24-1.69)	
3 - 6 years	1,939	51.1	(48.8-53.3)	48.7	(46.1-51.4)	39.7	(37.2-42.2)	11.6	(10.0-13.4)	2.08	(1.79-2.41)	
7 - 10 years	1,718	42.6	(40.4-44.8)	41.3	(38.9-43.8)	45.7	(43.3-48.1)	13.0	(11.4-14.8)	1.52	1.31-1.78)	
11 - 13 years	1,289	42.4	(40.1-44.7)	36.9	(34.0-40.0)	46.5	(43.1-49.8)	16.6	(14.3-19.2)	1.36	(1.15-1.60)	
14 - 17 years	1,864	50.7	(49.5-52.2)	32.5	(30.2-34.8)	56.1	(53.6-58.8)	11.4	(9.9-13.1)	1(Referenz)		
Region												.355
East	2,881	51.7	(49.2-54.2)	40.3	(38.3-42.4)	50.1	(47.9-52.2)	9.6	(8.7-10.7)	1.06	(0.93-1-19)	
West	6,018	50.7	(49.2-52.2)	40.1	(38.5-41.7)	48.5	(46.8-50.2)	11.4	(10.5-12.4)	1(Referenz)		
Urbanity												.022
Rural area	1,985	50.5	(47.8-53.1)	36.5	(33.4-39.8)	54.5	(51.0-58.0)	8.9	(7.1-11.1)	0.83	(0.69-1.00)	
Small city	2,314	50.9	(48.3-53.5)	42.0	(39.0-45.0)	47.3	(44.6-49.6)	10.8	(9.3-12.4)	1.10	(0.92-1.30)	
Medium-sized city	2,561	50.7	(48.2-53.3)	41.6	(39.4-43.8)	47.3	(45.2-49.4)	11.1	(10.0-12.4)	1.07	(0.91-1.24)	
Large city	2,039	51.2	(48.4-53.9)	39.1	(36.8-41.5)	47.9	(45.1-50.8)	12.9	(11.2-14.9)	1(Referenz)		
Migrant background												.954
Yes	1,071	41.4	(38.8-44.2)	35.8	(32.6-39.1)	44.1	(40.5-47.8)	20.1	(17.5-22.9)	0.99	(0.82-1.21)	
No	7,788	52.8	(51.5-54.1)	40.9	(39.4-42.3)	49.5	(48.0-51.1)	9.6	(8.8-10.4)	1(Referenz)		
Missing	40											
Social status												.157
Low	2,249	47.1	(45.0-49.2)	39.1	(36.6-41.7)	47.0	(44.4-49.7)	13.9	(12.1-15.8)	0.91	(0.78-1.07)	
Middle	4,083	51.2	(49.7-52.7)	39.9	(37.9-41.9)	50.4	(48.3-52.5)	9.7	(8.6-10.8)	0.90	(0.80-1.00)	
High	2,403	55.1	(53.0-57.3)	42.5	(40.4-44.7)	47.6	(45.4-49.8)	9.9	(8.5-11.4)	1(Referenz)		
Missing	164											
Parents’ rated subjective health status												< .001
Excellent	3,261	47.2	(45.4-48.9)	38.2	(36.1-40.3)	51.7	(49.6-53.8)	10.1	(9.1-11.2)	0.71	(0.58-0.88)	
Good	4,840	52.1	(50.6-62.9)	41.2	(39.4-42.9)	47.1	(45.3-49.0)	11.7	(10.5-13.0)	0.89	(0.73-1.07)	
Moderate/Bad/Very Bad	798	60.0	(49.5-52.2)	41.7	(37.9-45.6)	46.7	(42.8-50.7)	11.6	(9.3-14.3)	1(Referenz)		

n unweighted, % weighted.

**Figure 1:**
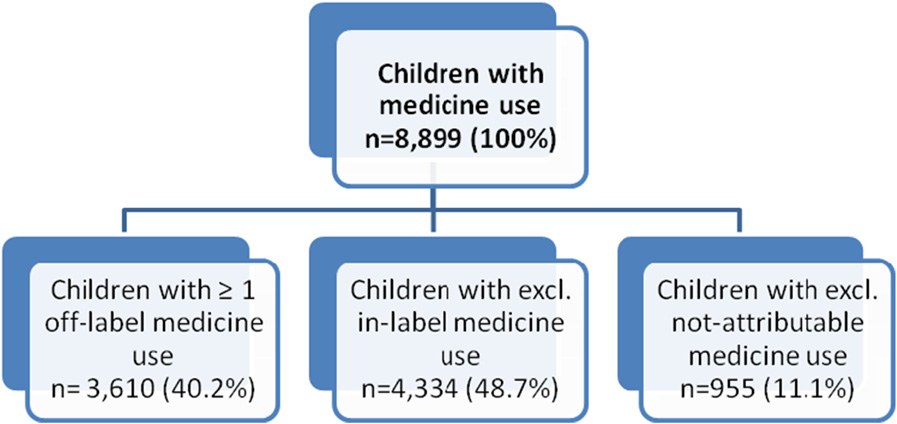
Prevalence of children with off-label-, in-label-, and not-attributable medicine use, KiGGS 2003–2006.

Among the study subjects 3,610 (40.2% 95% CI 38.8-41.5%) boys and girls use one or more preparations off-label. 4,334 children and adolescents use all their preparations exclusively in-label (48.7% 95% CI 47.3-50.2%). For 11.1% (95% CI 10.3-12.0%) of children with drug use (n = 955), it was not possible to identify in- or off-label use for any of their medications (Figure 1).

Regarding off-label medicine use statistically significant differences can be observed with respect to sex and age. Boys have a higher prevalence rate than girls (41.4 vs. 38.9%). Those below 2 years of age and those 14 years of age or older use significantly less off-label medication compared to those aged between 3 and 13 years. There are no significant differences according to region of residence and urbanity. Children and adolescents from families without migration background and from families with higher social status have a higher off-label medicine use, but the differences are only significant for migration background. Boys and girls whose parents indicated an excellent health status of their children receive significantly less off-label medication compared to those with a moderate/bad/very bad health status (Table 1).

The prevalence rate of children exclusively using in-label medicines, amounts to 48.7%. Exclusive in-label medicine use is significantly higher e.g. in girls, in the age groups 0 to 2 and 14 to 17 years, and in rural areas. Children with an excellent parents’ rated health status use their medication also more often exclusively in-label (Table 1).

For 955 participants (11.1%) the reported medicines could not be allocated with respect to in- or off-label medicine use, because information regarding the preparations was unspecific. Those children are significantly more often boys than girls (12.4 vs. 9.8%), and less often aged 0 to 2 years. Children with migration background or living in families with a lower social status have a significantly higher rate of not-attributable medicine use. There are no significant differences in the prevalence rate of not-attributable medicine use according to parents’ rated health status (Table 1).

In the logistic regression model sex, age, urbanity and parents’ rated health status are independent determining factors for the probability of off-label medicine use. Boys have a significantly higher OR than girls, and children up to 13 years of age have a higher OR than adolescents (14 to 17 years). Living in rural areas is associated with a significantly lower OR compared to living in large cities. If the parents rated the health status of their children as excellent the OR is significantly lower compared to a moderate/bad/very bad health status (Table 1).

### Spectrum of off-label use

8,899 study participants had taken 14,588 preparations resulting in an average of 1.62 (95% CI 1.59-1.66) preparation per drug user.

**Figure 2:**
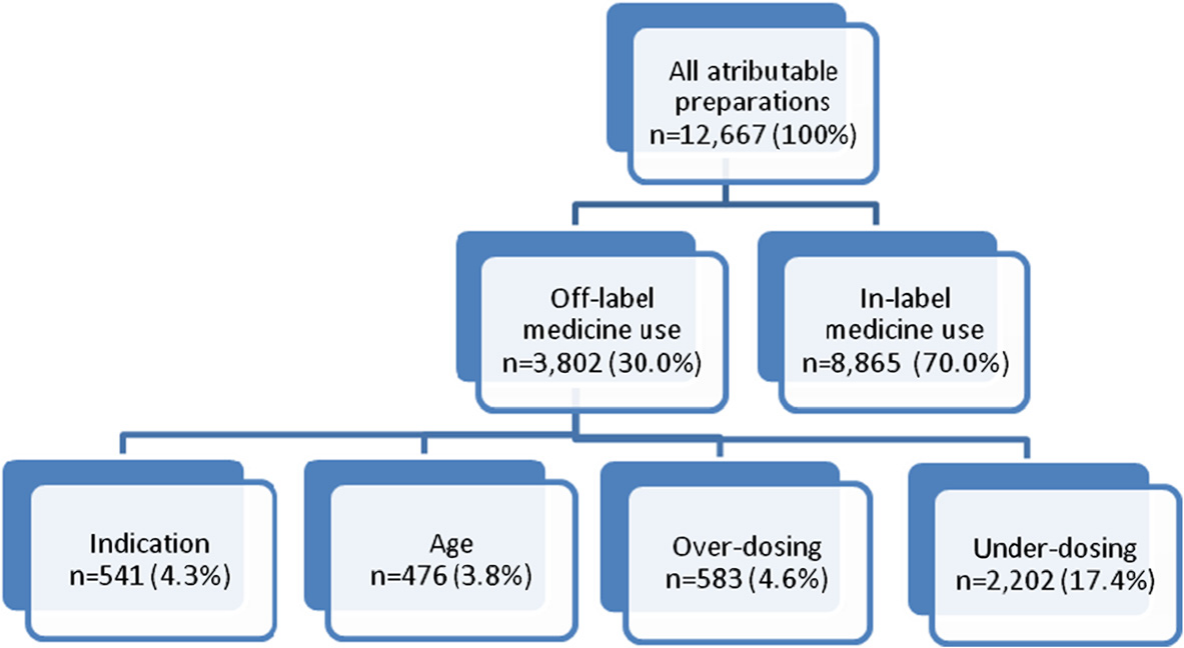
Drug use by in-label and off-label medicine use (only attributable preparations), KiGGS 2003–2006.

Among 14,588 preparations recorded, 3,802 are classified as “off-label”, 8,865 as “in-label” and 1,921 (13.2%) as “not-attributable”. After excluding not-attributable preparations there are 12,667 preparations left for further analyses. Among these 12,667 medicines 70.0% are used in-label. Within the remaining 30% off-label use, under-dosing (17.4%) is the most frequent category, followed by over-dosing (4.6%), indication (4.3%), and age (3.8%) (Figure 2).

**Figure 3:**
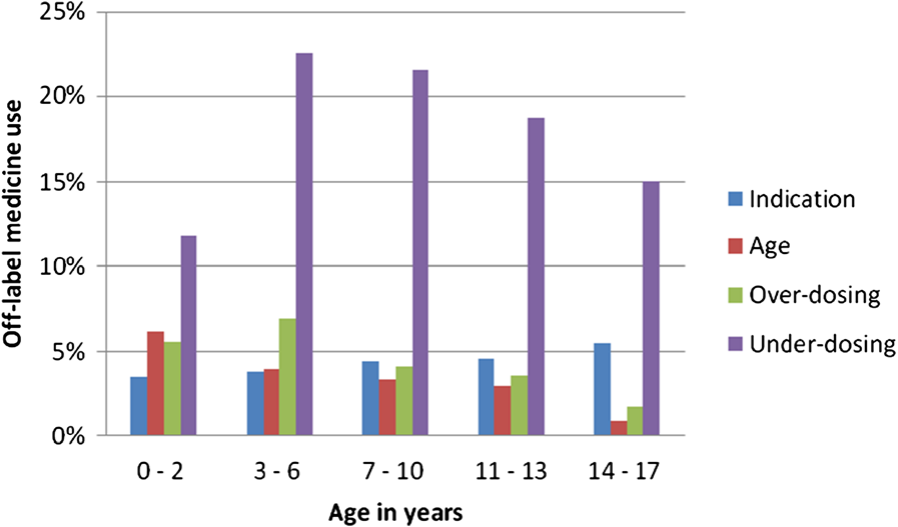
Off-label medicine use by off-label strata and age groups, KiGGS 2003–2006.

In Figure 3 the distribution of off-label medicine use according to age groups is illustrated. In all age groups under-dosing is the main reason for off-label use (0–2 years 11.8%, 3–6 years 22.6%, 7–10 years 21.5%, 11–13 years 18.7%, and 14–17 years 15.0%). Age is the second most common reason for off-label medicine use in the age group 0–2 years (6.2%) and over-dosing in the age group 3 – 6 years (6.9%). Indication is the second most common reason in all other age groups (7–10 years: 4.4%, 11–13 years: 4.6%, and 14–17 years: 5.5%).

61.2% of the used preparations are prescribed by a physician or a non-medical practioner, 38.8% are self-medicated (OTC 24.7%, 14.1% from other sources). Looking at off-label medicine use according to origins of medicines (prescribed, OTC, other sources) we find the following results: about 30% of prescribed as well as OTC products or preparations from other sources are used off-label. Under-dosing is the most common off-label use in all categories (prescribed medicines: 16.1%, OTC: 19.3%, medicines from other sources: 20.2%). Under-dosing is significantly more frequent in OTC products and preparations from other sources compared to prescribed medicines. In contrast, off-label indication is significantly higher in prescribed medication than in OTC products and preparations from other sources (Table 2).

**Table 2:** Off-label and in-label medicine use by origin of medicines, KiGGS 2003-2006

**Drug use categories**	Origin of medicines (n = 12,587*)								
	**Prescribed**			**OTC**			**Other sources**		
	**n**	**%**	**95% CI**	**n**	**%**	**95% CI**	**n**	**%**	**95% CI**
**Off-label medicine use**	**2,344**	**29.9**	**(28.9-30.9)**	**925**	**30.3**	**(28.7-31.9)**	**515**	**30.6**	**(28.5-32.9)**
➢ Indication	376	4.8	(4.3-5.2)	106	3.5	(2.9-4.2)	56	3.3	(2.6-4.3)
➢ Age	326	4.2	(3.7-4.6)	100	3.3	(2.7-4.0)	45	2.7	(2.0-3.6)
➢ Under-dosing	1,263	16.1	(15.3-16.9)	590	19.3	(18.0-20.8)	339	20.2	(18.3-22.2)
➢ Over-dosing	379	4.8	(4.4-5.3)	129	4.2	(3.6-5.0)	75	4.5	(3.6-5.6)
**In-label medicine use**	**5,508**	**70.1**	**(69.1-71.1)**	**2,129**	**69.7**	**(68.1-71.3)**	**1,166**	**69.4**	**(67.1-71.5)**

* Discrepancy to total n = 12,667 because of missing data (n = 80). OTC = Over the Counter.

In our study we also analyzed off-label medicine use according to ATC-classes/ATC-substance-groups. Overall off-label medicine use is highest (67.2%) for products belonging to the cardiovascular system (ATC-code C00) and lowest (13.3%) for products belonging to urogenital tract and sexual hormones (ATC-code G00). Off-label age and off-label indication is higher in the ATC- class C00 and in the ATC-class L00 (antineoplastic and immune modulating agents). In all other ATC-classes off-label under-dosing is dominating. This includes e.g. antibiotics (ATC-code J01) with 21.3% under-dosing (Table 3).

**Table 3:** Off-label and in-label medicine use by ATC-classes and off-label strata, KiGGS 2003-2006

**ATC-class**	**Off-label use**								**In-label use**		**Total**
	**Indication**		**Age**		**Over-dosing**		**Under-dosing**				
	**n**	**%**	**n**	**%**	**n**	**%**	**n**	**%**	**n**	**%**	**n**
**Total**	**541**	**4.3**	**476**	**3.8%**	**583**	**4.6**	**2,202**	**17.4**	**8,865**	**70.0**	**12,667**
**A00 ALIMENTARY TRACT and METABOLISM**	**140**	**4.8**	**113**	**3.9%**	**86**	**2.9**	**654**	**22.4**	**1,933**	**66.1**	**2,926**
A01 Stomatological preparations	47	2.8	80	4.7%	41	2.4	308	18.2	1215	71.9	1691
A03 Antispasmodic and anticholinergic agents and propulsives	6	4.7	3	2.4	8	6.3	18	14.2	92	72.4	127
A07 Antidiarrheals. Intestinal Antiinflammatory/Antiinfektive Agents	5	4.9	7	6.8	6	5.8	11	10.7	74	71.8	103
A11 Vitamins	35	5.1	10	1.4	18	2.6	218	31.5	411	59.4	692
A12 Mineral Supplements	42	22.5	4	2.1	6	3.2	84	44.9	51	27.3	187
A14 Anabolic Agents for Systemic Use	0	0	0	0	0	0	0	0	2	100	2
**B00 BLOOD and BLOOD FORMING ORGANS**	**0**	**0**	**4**	**5.5**	**3**	**4.1**	**17**	**23.3**	**49**	**67.1**	**73**
**C00 CARDIOVASKULAR SYSTEM**	**3**	**5.2**	**11**	**19.0**	**3**	**5.2**	**22**	**37.9**	**19**	**32.8**	**58**
C01 Cardic Therapy	0	0	0	0	0	0	3	30.0	7	70.0	10
C05 Vasoprotectives	1	4.0	0	0	1	4.0	17	68.0	6	24.0	25
C07 Beta Blocking Agents	1	10.0	7	70.0	0	0	1	10.0	1	10.0	10
**D00 DERMATOLOGICALS**	**69**	**4.4**	**42**	**2.7**	**30**	**1.9**	**344**	**21.8**	**1,094**	**69.3**	**1579**
D01 Antifungals for Dermatological Use	7	6.1	1	0.9	5	4.3	21	18.3	81	70.4	115
D02 Emollients and Protectives	4	1.6	4	1.6	4	1.6	29	11.9	203	83.2	244
D03 Preparations for Treatment of Wounds and Ulcers	17	5.3	2	0.6	0	0	3	0.9	297	93.1	319
D04 Antipruritics. incl. Antihistamines. Anesthetics etc.	1	0.6	1	0.6	1	0.6	46	28.6	112	69.6	161
D06 Antibiotics and Chemotherapeutics for Dermatological Use	10	11.6	1	1.2	2	2.3	49	57.0	24	27.9	86
D07 Corticosteroids. Dermatological Preparations	23	10.2	15	6.6	13	5.8	95	42.0	80	35.4	226
D10 Anti-Akne Preparations	2	1.8	0	0	1	0.9	28	25.5	79	71.8	110
D11 Other Dermatological Preparations	4	1.8	16	7.4	3	1.4	69	31.8	125	57.6	217
**G00 GENITO URINARY SYSTEM and SEX HORMONES**	**52**	**10.5**	**2**	**0.4**	**4**	**0.8**	**8**	**1.6**	**431**	**86.7**	**497**
G03 Sex Hormones and Modulators of the Genital System	48	10.6	1	0.2	3	0.7	2	0.4	398	88.1	452
**H00 SYSTEMIC HORMONAL PREPARATIONS. excl. SEX HORMONES and INSULINS**	**6**	**2.0**	**0**	**0**	**7**	**2.4**	**29**	**9.8**	**253**	**85.8**	**2,,95**
H03 Thyroid Therapy	1	0.4	0	0	5	2.0	28	11.4	212	86.2	246
**J00 ANTIINFECTIVES for SYSTEMIC USE**	**25**	**6.7**	**5**	**1.3**	**21**	**5.6**	**71**	**19.0**	**252**	**67.4**	**374**
J01 Antibiotics for Systemic Use	25	7.5	4	1.2	21	6.3	71	21.3	212	63.7	333
**L00 ANTINEOPLASTIC and IMMUNMODULATING AGENTS**	**8**	**9.4**	**7**	**8.2**	**5**	**5.9**	**24**	**28.2**	**41**	**48.2**	**85**
L03 Immunostimulants	8	11.3	6	8.5	4	5.6	23	32.4	30	42.3	71
**M00 MUSCULO-SKELETAL SYSTEM**	**11**	**2.0**	**31**	**5.6**	**8**	**1.5**	**47**	**8.5**	**454**	**82.4**	**551**
M01 Antiinflammatory and Antirheumatic Products	2	0.8	16	6.4	6	2.4	26	10.4	200	80.0	250
M02 Topical Products for Joint and Muscular Pain	7	2.5	14	5.0	1	0.4	16	5.7	242	86.4	280
**N00 NERVOUS SYSTEM**	**28**	**2.6**	**43**	**3.9**	**11**	**1.0**	**93**	**8.5**	**921**	**84.0**	**1,096**
N02 Analgesics	11	1.4	30	3.9	5	0.6	66	8.5	664	85.6	776
N06 Psychoanaleptics	7	3.8	2	1.1	0	0.0	9	4.9	167	90.3	185
**R00 RESPIRATORY SYSTEM**	**94**	**2.4**	**202**	**5.2**	**334**	**8.6**	**786**	**20.2**	**2477**	**63.6**	**3,893**
R01 Nasal Preparations	13	1.3	55	5.5	18	1.8	60	6.1	845	85.3	991
R02 Throat Preparations	2	1.3	2	1.3	13	8.7	9	6.0	124	82.7	150
R03 Anti-Asthmatics	20	3.5	46	8.0	16	2.8	72	12.6	419	73.1	573
R04 Chest Ointments. Inhalatives	3	1.0	35	11.3	0	0.0	108	34.7	165	53.1	311
R05 Cough and Cold Preparations	42	2.6	54	3.3	278	16.9	476	28.9	797	48.4	1647
R06 Antihistamines for Systemic Use	13	6.1	10	4.7	9	4.2	60	28.3	120	56.6	212
**S00 SENSORY ORGANS**	**20**	**10.0**	**2**	**1.0**	**10**	**5.0**	**60**	**30.0**	**108**	**54.0**	**200**
S01 Ophthalmologicals	19	13.4	2	1.4	2	1.4	47	33.1	72	50.7	142
S02 Otologicals	1	1.9	0	0	7	13.5	11	21.2	33	63.5	52
**V00 VARIOUS**	**5**	**2.3**	**1**	**0.5**	**6**	**2.8**	**23**	**10.6**	**183**	**83.9**	**218**
V03 All Other Therapeutic Products	3	3.0	0	0	4	4.0	18	18.2	74	74.7	99
V06 General Nutrients	0	0	1	2.0	2	4.0	5	10.0	42	84.0	50
**Z00 HOMOEOPATHICS**	**80**	**10.0**	**11**	**1.4**	**55**	**6.9**	**23**	**2.9**	**631**	**78.9**	**800**

Off-label medicine use stratified by ATC-classes shows differences in the various age groups. Until the age of 2 years the most frequently used preparations are medications for sensory organs (ATC-code S00), followed by medicines for the respiratory system (ATC-code R00) and for antiinfectives for systemic use (ATC-code J00). In the age of 3 to 10 years preparations for alimentary tract and metabolism (ATC-code A00), for sensory organs (ATC-code S00) and for respiratory system (ATC-code R00) dominate off-label medicine use. In adolescents and youths (11 to 17 years) most often used off-label medicines are preparations of the ATC-class A00 (alimentary tract and metabolism) followed by ATC-class D00 (dermatologicals) and S00 (sensory organs) (Table 4).

**Table 4:** Off-label medicine use by ATC-classes and age groups, KiGGS 2003-2006

**ATC-class**	**0-2 years**	**3-6 years**	**7-10 years**	**11-13 years**	**14-17 years**
	**n (%)**	**n (%)**	**n (%)**	**n (%)**	**n (%)**
**A00 Alimentary tract and metabolism**	328 (21.3)	255 (49.5)	172 (50.0)	108 (47.8)	130 (43.0)
**B00 Blood and blood forming organs**	2 (13.3)	1 (14.3)	2 (50.0)	10 (52.6)	9 (32.1)
**C00 Cardiovascular system**	3 (100)	2 (40.0)	9 (64.3)	9 (90.0)	16 (61.5)
**D00 Dermatologicals**	102 (26.6)	106 (30.1)	110 (31.0)	75 (37.1)	92 (32.1)
**G00 Genito urinary system and sex hormones**	4 (66.7)	1 (10.0)	6 (50.0)	2 (25.0)	53 (11.5)
**H00 Systemic hormon. preparations excl. sex hormones and insulins**	3 (14.3)	11 (19.3)	7 (13.7)	7 (9.5)	14 (15.2)
**J00 Antiinfectives for systemic use**	29 (32.2)	41 (36.6)	18 (30.5)	15 (31.9)	19 (28.8)
**L00 Antineoplastic and immunmodulating agents**	7 (70.0)	16 (57.1)	8 (42.1)	6 (42.9)	7 (50.0)
**M00 Musculo-skeletal system**	13 (25.5)	17 (23.3)	18 (16.8)	21 (18.9)	28 (13.4)
**N00 Nervous system**	20 (12.7)	21 (20.2)	51 (25.5)	39 (16.5)	44 (11.1)
**P00 Antiparasitic products insecticides and repellents**	1 (33.3)	1 (20.0)	1 (14.3)	0	
**R00 Respiratory system**	356 (39.1)	493 (40.4)	288 (35.7)	146 (30.7)	133 (27.7)
**S00 Sensory organs**	30 (56.6)	30 (42.3)	13 (61.9)	11 (36.7)	8 (32.0)
**V00 Various**	16 (16.3)	6 (25.0)	7 (16.7)	1 (5.6)	5 (13.9)
**Z00 Homoeopathics**	41 (23.2)	47 (20.3)	37 (19.2)	22 (20.6)	22 (23.9)
**TOTAL n = 12,667**	954 (27.1)	1048 (37.3)	747 (33.5)	473 (29.8)	580 (23.0)

### Quality control

The re-assessment of a 5% random-sample by a second reviewer revealed a similar distribution for off-label medicine use. Inter-rater reliability was found to be “good” (Kappa = 0.655).

## Discussion

### Principle findings

Looking at the population level, the prevalence rate of off-label medicine use in children/adolescents, who took at least one preparation amounts to 40.2%. Off-label medicine use is statistically significantly higher in boys and lower in adolescents (14–17 years). There are no significant differences according to region, urbanity, migrant background, and social class. A better parents-rated health status of their children is associated with a lower probability of off-label medicine use. Looking at the preparation level, 70% of all attributable preparations are used in-label and 30% off-label. The spectrum of off-label medicine use is dominated by under-dosing (17.4%), followed by over-dosing (4.6%), indication (4.3%), and age (3.8%). Furthermore we found that under-dosing is the most common reason for off-label-use in prescribed medicines as well as in self-medication.

### Strengths and limitations

The KiGGS study provides representative population-based data on drug use among children and adolescents. The data have been obtained by physicians administered structured interviews including a population of more than 17,000 children and adolescents. Data obtained in this study are independent from the provision of health care and provide information about the “real” use of medicines in the population. In contrast, studies utilizing prescription data, health insurance data, secondary care data, or medication sales data are unable to consider if patients actually took the medicines and thus do not represent the real use. Prescribed or OTC medication may be taken in various quantities, at a different point of time or by a different person. In this study, information on drug use was obtained by face-to-face interviews, which contributes to reliable data on actual use of medication.

The findings of this study are generalizable with respect to off-label use of medicine among children and adolescents aged 0–17 years in Germany. Whereas the analysis of prescription data only allows conclusions about off-label prescription but not about off-label medicine use, and the data on sales of OTC medicine does not give information on user patterns, the KiGGS-data provide information about the use and off-label use of all medications including OTC products and medicines from other sources. In addition KiGGS provides information on the associations between socio-economic as well as health-relevant indicators and off-label medicine use. Thus the results of our study are unique in Germany and international.

However, this study also has several limitations. Information regarding the use of medication collected were provided by parents and/or supplemented with information from the adolescents. Therefore a recall-bias has to be considered. In order to minimise this effect parents and children/adolescents were asked to bring along packages from all products recently used. But only for about 30% of recorded preparations this was done. Even though this proportion is low, about 87% of all preparations could be assessed according to off-label use. This underlines that the information relying on memorising preparations used in the last seven days are of a sufficient validity.

Memory problems are mainly related to dosing information, particularly if the product is not used regularly. The high proportion of under-dosing may indicate this problem, however, previous studies evaluating off-label medicine use and considering the dosing came to similar results as our study [11,12].

Another limitation can arise from the self-reported reason for treatment (indication). This may be inadequate as there may be problems regarding the communication between the consulted physicians and parents, thus leading to a misunderstanding of the indication. Our results support this assumption, as they show that children with migration background or from families with a lower social status have a significantly higher prevalence rate of exclusively not-attributable medicine use.

### Prevalence and determinants of off-label medicine use

Population representative data regarding off-label medicine use in children and adolescents which are independent from any health care provisions are rare in the literature. The prevalence of off-label use among medicine users identified in our study is 40.2%. This differs from the results of a Scottish study (Ekins-Daukes et al.) where prevalence is found to be 10.6% [11]. Cuzzolin et al. reviewed international studies and report unlicensed and off-label rates in ambulatory care between 13.2% and 29%, in paediatric wards between 18% and 60% and in neonatal units between 14% and 63% [12]. Another international literature review by Pandolfini and Bonati reports that rates for off-label medicine use vary between 11% and 80%. Higher rates are seen in younger patients and in hospital settings [17]. However, comparing these studies with our data is difficult as there are differences in respect to the study design, study population and periods of observation.

In our analysis children who were aged 3 to 13 years show higher off-label medicine use compared to those drug users aged 0–2 years and 14–17 years respectively. This is contradictory to the findings of previously published studies where highest rates for off-label medicine use are in neonates [4,11,18,25-27]. The main reason for these differences may be related to differences in the study population. While the KIGGS study population includes no hospitalized and mainly healthy children, previously published studies include hospitalised and intensive care patients.

### Spectrum of off-label medicine use

In our study 30% of all attributable products are used off-label. This proportion is above the previously reported data (13.2%) in Germany (Buecheler et al.). However, Buecheler et al. do not supply information regarding dosing and indication [4]. Consequently their data are underestimating the prevalence which may explain the difference to our findings. T’Jong et al. report in their study from the Netherlands that 44% of all prescriptions in a paediatric ward are off-label [28]. In ambulant patients this rate amounts to 23% and is slightly lower compared to the findings in our study [29].

Under-dosing is the most frequent reason for off-label medicine use in our study, accounting for more than half of all off-label medications. Similar findings were previously reported from Scotland and Brazil [11,30].

Off-label medicine use such as under- and over-dosing could bear the risk of potential health hazards. Inappropriate dosing is of particular concern for antibiotic use with respect to the development of resistances [31,32] but also regarding adverse drug reactions (ADRs) [33-35]. If medication is under-dosed possibly no therapeutic benefit but a risk for ADRs could occur, as those often are independent of the dose. In our study 36% of antibiotics are used off-label, of those 58% are under-dosed. Similar findings are reported from Porta et al. who found that under-dosing of antibiotics is the most common reason for off-label use in the UK and in Italy [30]. A Scottish study of Ekins-Daukes et al. reports that the number of children who had been prescribed antibiotics of a less than recommended dose increases with age from 11.8% in the age group 0–4 years to 30.0% in the age group 12–16 years [36]. The problem is also highlighted in a historic review from England by Ahmed et al. who is concerned that many antibiotics used in children are under-dosed [37].

Menson et al. investigate the use of anti-retrovirals in UK and Irish children and identify that children have frequently been under-dosed with anti-retrovirals over the observed 9 year period [38]. Major reasons identified are inconsistent dosage strategies or failure to respond to growth, especially at extremes of weight bands. As children grow, drug doses need regular adjustment and failure to do so may reduce the benefits of treatment.

Kazouini et al. analyse Paracetamol prescription issued in 2006 to Scottish children and find that 13.3% of prescriptions are under-dosed and 4.4% are over-dosed [39]. In a similar study the authors show that prescribing of antibiotics below the recommended doses is more frequent than the prescribing of doses which are above official recommendations [36]. This implies that the overall large extent of under-dosing regarding the use of drugs in children is not exclusive to Germany. However, most of the previously published data come from hospital or ambulatory care settings.

In our setting which was independent from health care provisions there is a large amount of self-medication (38.8%). This is not surprising and has previously already been reported in a study by Du and Knopf [40]. In the present study, within self-medication, off-label medicine use amounts to about 30% (OTC 30.3%, other sources 30.6%). Our data are representative for Germany and we could not identify any published studies from other countries related to off-label medicine use of self-medication, including OTC products. In self-medication as well as in prescribed medicine under-dosing is the most frequent reason for off-label medicine use, but in self-medication under-dosing is significantly higher. Here further studies are needed to investigate the reasons why there is such a high level of under-dosing of children’s medication. From a pharmacological point of view this doesn’t make sense because adverse reactions can occur at any dose whereas the desired effect is always related to the therapeutic dose. Furthermore, although the relationship is not significant in our analysis, the fact that children without migration background and with higher social status take more off-label medicine throws up the question why these groups of children are more likely to be exposed to off-label medicine use. A relationship between socio-economic factors and self-medication use was reported previously from various countries [40-43]. A study of Du and Knopf shows that parents with higher social economic status tend to utilize more self-medication for their children [40].

## Conclusions

Our data show that there is a high level of off-label medicine use in the general paediatric population and that under-dosing is the most frequent reason for off-label use. This could put drug users at the risk of side effects without a therapeutic effect. Particularly for antibiotics the development of resistances is fostered when too low doses are given.

With respect to rational use of medicines the correct dose should be given to ensure safety and effectiveness of pharmacotherapy. Further studies are needed to investigate why there is inadequate dosing.

Understanding the prevalence, determinants and spectrum of off-label medicine use can help developing prevention strategies. Further studies are needed to investigate the reasons for off-label medicine use as it might be due to lack of knowledge in paediatric pharmacotherapy or the absence of appropriate paediatric medication or based on empiricism.

The high amount of off-label medicine use in self-medication needs further research to identify whether this is a particular German phenomenon or whether similar patterns can be observed in other countries as well.

In addition to appropriate clinical trials for licensing of paediatric medicines, education of health care professionals but also of parents and carers about the rational and correct use of medicines is needed. This will ensure the most appropriate use of medicines in the paediatric population so that drug use can be based on well-grounded information.

## Competing interest

The authors declare that they have no competing interests.

## Authors’ contributions

HK coordinated the conceptualization and conduction of the project, performed the statistical analysis, wrote and finalised the manuscript. IW assisted in analysing the data and interpreting the results, writing and finalising the manuscript. GS and WZ conducted the data collection, provided the literature review and assisted in analysing the data and interpreting the results. WR had provided the initial input and reviewed the manuscript. AN provided specific knowledge, assisted in the conceptualization of the study, and contributed writing to the manuscript. All authors read and approved the final manuscript. HK is the guarantor for the study.

## Pre-publication history

The pre-publication history for this paper can be accessed here:

http://www.biomedcentral.com/1471-2458/13/631/prepub
